# Chronic clinical signs of upper respiratory tract disease associate with gut and respiratory microbiomes in a cohort of domestic felines

**DOI:** 10.1371/journal.pone.0268730

**Published:** 2022-12-01

**Authors:** Holly Kristin Arnold, Rhea Hanselmann, Sarah M. Duke, Thomas J. Sharpton, Brianna R. Beechler

**Affiliations:** 1 Department of Microbiology, Oregon State University, Corvallis, Oregon, United States of America; 2 Carlson College of Veterinary Medicine, Oregon State University, Corvallis, Oregon, United States of America; 3 College of Veterinary Medicine, Western University of Health Sciences, Pomona, California, United States of America; 4 Department of Statistics, Oregon State University, Corvallis, Oregon, United States of America; 5 Department of Biomedical Sciences, Oregon State University, Corvallis, Oregon, United States of America; Montana State University, UNITED STATES

## Abstract

Feline upper respiratory tract disease (FURTD), often caused by infections etiologies, is a multifactorial syndrome affecting feline populations worldwide. Because of its highly transmissible nature, infectious FURTD is most prevalent anywhere cats are housed in groups such as animal shelters, and is associated with negative consequences such as decreasing adoption rates, intensifying care costs, and increasing euthanasia rates. Understanding the etiology and pathophysiology of FURTD is thus essential to best mitigate the negative consequences of this disease. Clinical signs of FURTD include acute respiratory disease, with a small fraction of cats developing chronic sequelae. It is thought that nasal mucosal microbiome changes play an active role in the development of acute clinical signs, but it remains unknown if the microbiome may play a role in the development and progression of chronic clinical disease. To address the knowledge gap surrounding how microbiomes link to chronic FURTD, we asked if microbial community structure of upper respiratory and gut microbiomes differed between cats with chronic FURTD signs and clinically normal cats. We selected 8 households with at least one cat exhibiting chronic clinical FURTD, and simultaneously collected samples from cohabitating clinically normal cats. Microbial community structure was assessed via 16S rDNA sequencing of both gut and nasal microbiome communities. Using a previously described ecophylogenetic method, we identified 136 and 89 microbial features within gut and nasal microbiomes respectively that significantly associated with presence of active FURTD clinical signs in cats with a history of chronic signs. Overall, we find that nasal and gut microbial community members associate with the presence of chronic clinical course, but more research is needed to confirm our observations.

## Introduction

Feline upper respiratory tract disease (FURTD), most often caused by infectious etiologies, is a highly prevalent multifactorial syndrome that affects felid populations worldwide. Due to the highly contagious nature of infectious FURTD pathogens, infectious FURTD is most prevalent anywhere cats live in groups [1], and thus FURTD is a major burden on animal shelters [2]. Clinical signs of infectious FURTD include ocular or nasal discharge, sneezing, epistaxis, and severe ocular, nasal, or oral pathology including inflammation and ulceration of mucous membranes and corneas [3, 4]. Even though morbidity greatly surpasses mortality in natural infection course [5], infectious FURTD can increase euthanasia rates in shelters [6] by increasing time to adoption, contributing to overcrowding, and shunting resources into costly clinical treatments and isolation/sanitization protocols [5]. Understanding the etiology of infectious FURTD is thus a priority in feline medicine to better mitigate the negative consequences of this disease.

Unfortunately, both FURTD etiology and pathophysiology remain difficult to unravel in part because of the complexity of the disease. At least six primary pathogens have been identified as causing signs of disease, including viral (Feline Herpes Virus 1, Feline Calicivirus), bacterial (*Bordetella bronchiseptica*, *Chlamydophila felis*, *Mycoplasma* species), and less commonly fungal (*Cryptococcus neoformans*) organisms. Infection with a primary agent is thought to proceed a secondary change in underlying microbial community composition, both of which likely contribute to clinical signs. Using culture-independent 16S sequencing, prior work has shown that nasal microbial communities are indeed shaped by the presence of primary FURTD pathogens during acute infection [7]. Some, but not all, cats will go on to develop a chronic disease course from presence of latent infection (i.e. Feline Herpes Virus 1), or an immune-mediated response to infection [5]. It remains unknown, however, if secondary microbial changes persist in cats with chronic clinical disease and if such changes contribute to persistence of clinical signs.

We reasoned that cats with long-term FURTD signs may develop a chronicity in clinical course due to unique interactions between primary FURTD pathogen and persistent changes within the respiratory microbiome. For example, widely reported detection ranges (9–89%) for Feline Herpes Virus 1 (FeHV-1) in cats with clinical signs consistent with FURTD [8–17], as well as identification of FeHV-1 in cats suspected to be clinically normal (3–49%) [8, 12, 14, 15, 17, 18], begs the question on why some cats with primary etiological agents do not chronically present with clinical signs while others do. We hypothesized that cats identified as having chronic clinical manifestations consistent with infectious FURTD signs may have upper respiratory microbiome changes compared to cats without chronic clinical signs.

Recent studies increasingly note the presence of a gut-respiratory axis, resulting in a dynamic interplay between gut and airway microbial communities modulated by shared mucosal immunity [19–21]. Changes within the gut microbiome may happen secondarily to upper respiratory tract disease as a result of systemic inflammation [22], or may be a primary cause leading to signs of upper respiratory tract disease. For example, exposure to dust microbial communities altered gut microbiome community structure in a mouse model, and consequently protected hosts from airway viral infection [23]. Disrupted gut communities can influence immune responses at distal sites [24, 25], and microbial diversity exposure has been inversely correlated to allergy, asthma and allergic rhinitis development in humans [26–28]. Therefore, we hypothesized that cats with chronic FURTD clinical signs may also have changes that are apparent within their gut microbiome.

Culture-independent 16S rDNA sequencing of the cat upper respiratory microbiome has revealed a rich and variable respiratory community that was incompletely documented by culture techniques alone [7]. Significant variability in upper respiratory microbiome composition was observed both within and among cats with and without clinical signs, as well as across age classes, sex, and breed groups [7, 29]. The high variation of respiratory compositions in prior work resulted in difficulty distinguishing key microbial taxa which consistently differed between control and FURTD subjects [7], some of which may have been driven by differing environmental factors [29, 30]. We reasoned that a large contributing factor to nasal and gut microbiome communities is the metacommunity to which the cat is exposed (i.e., household). Thus, we developed a study design where each household was recruited on the basis of having at least one cat with a history of chronic upper respiratory tract signs, as well as also sampling apparently healthy cats in the same household when possible.

Our objective was to further characterize the etiology of FURTD in cats with and without chronic clinical signs. We show that markers suggestive of inflammation significantly differ in cats with FURTD signs compared to those without. We then apply a novel ecophylogenetics technique [31, 32] in order to discover monophyletic microbial clades and individual microbial biomarkers that exhibit differential ecological distributions across cats based on disease status. Last, we develop a novel bootstrapping approach to determine whether microbiome features significantly associated with clinical signs are phylogenetically clustered or overdispersed.

## Materials and methods

### Population recruitment and study design

We selected eight households where each household was recruited on the basis of having at least one cat with a history of chronic upper respiratory tract signs of at least one year duration. When possible, we also sampled apparently healthy cats in the same household. Clients were recruited from Oregon State University (OSU) in Corvallis, Oregon, as well as from community members within the broader Corvallis area. All animal work was approved by the OSU Institutional Animal Care and Use Committee (IACUC) under ACUP protocol #5102. A written consent form was approved by IACUC before use and written informed consent was obtained from all animal owners prior to participation.

All participating cats were housed primarily indoors and had proof of current rabies vaccination. Cats were required to have lived in the same household for at least one year to account for environment-associated variation in microbiome communities [29]. A thorough clinical history and physical examination performed by a veterinarian and baseline diagnostics were obtained to exclude cats with concurrent disease. Cats with recent (i.e., < 21 days) history of vaccination with modified live organisms (parenteral or intranasal), treatment with antibiotics, anti-inflammatories, or immunosuppressive drugs (i.e., < 3 months), or had a known history of concurrent non-respiratory illness were excluded (S1 Table). Cats were also excluded if the presence of systemic disease was detected in baseline diagnostics (i.e., end-stage chronic kidney disease, hepatic, intestinal, or other signs of significant systemic illness), or if they were positive for Feline Leukemia Virus (FeLV), Feline Immunodeficiency Virus (FIV), or for heartworm (*Dirofilaria immitis*) during infectious disease screening.

### Classification of cases and controls

All cats were examined by a veterinarian to confirm the presence or absence of clinical signs. For analytical purposes, each clinical sign observed on physical exam was encoded as present if it was a sign consistent with infectious FURTD and absent otherwise for each system examined. This resulted in a matrix of systems-by-subject where every entry was scored as a 1 if abnormal and consistent with infectious FURTD and a 0 otherwise. For example, “dental tartar” was entered as a 0, because, while an abnormal physical finding, it is not a finding consistent with infectious FURTD. Cats were classified into the FURTD case category if they had a history of chronic FURTD signs and/or the presence of at least one clinical sign consistent with infectious FURTD was observed on veterinary physical examination. Cats were classified as controls if they did not meet these criteria. Clinical signs that were encoded as consistent with FURTD included ocular (bilateral serous or mucopurulent ocular discharge, conjunctivitis, chemosis, keratitis, corneal ulceration, and anterior uveitis), nasal (bilateral serous to mucopurulent nasal discharge, sneezing, and rhinitis), and oropharyngeal (stomatitis, glossitis, faucitis) clinical signs.

### Sample collection and processing

Cats were sampled between January and March 2019. Cohabiting cats were examined on the same day and in their residence to minimize stress-induced responses. Samples for nasal microbiome sequencing were obtained by pooling separate conjunctival, nasal, and oropharyngeal sterile synthetic swabs (Copan®, FLOQSwabsTM, Brescia, Italy), and were placed in 5mL of sterile phosphate-buffered saline (PBS) and stored at -80°C until extraction [7, 30, 33]. From each cat, 4 mL of blood was collected via peripheral venipuncture for a chemistry panel (serum), complete blood count (whole blood in EDTA), and FeLV/FIV/heartworm testing (serum). Samples for gut microbiome, intestinal (i.e. sugar flotation), and respiratory (i.e. Baermann) parasite screening [34] were collected using a fecal loop. All samples were stored at 4°C until processing within 24 hours of collection. Swabs were vortexed vigorously and 2mL aliquots of PBS were removed and stored separately. Both aliquots were centrifuged at 2500g for 20 minutes [30]. The resulting pellets were resuspended in 500μL PBS, and stored at -80°C until extraction. Original swabs and remaining PBS were submitted for primary FURTD pathogen testing.

### Baseline diagnostics

Baseline diagnostics included a chemistry panel, and complete blood count for all cats to identify inflammatory markers and screen for systemic disease (S1 Table). When samples were available, a urinalysis was performed to evaluate renal function and to screen for presence of uropathology, and a fecal sample was examined for intestinal and respiratory parasites. Serological evaluation for FeLV antigen, FIV antibodies, and heartworm antigen was performed in all cats (SNAP Feline Triple Test, IDEXX Laboratories Inc., Westbrook, Maine, USA). All cats were screened for primary bacterial and viral infectious FURTD pathogens using the Feline Respiratory Panel performed at the University of California Davis School of Veterinary Medicine Real-time PCR Research and Diagnostics Core Facility. All other baseline diagnostics were performed at Oregon Veterinary Diagnostic Laboratory at OSU Carlson College of Veterinary Medicine or submitted to IDEXX Laboratories Inc.

### 16S rDNA extraction and processing of gut and nasal microbiomes

All samples used for microbiome analyses were processed on the same day. At the time of DNA extraction, the swabs in PBS were removed from the freezer, thawed at 37°C, and vortexed vigorously for 15 seconds. The swab was removed, and the remaining PBS was centrifuged at 2500g for 20 minutes. The resulting pellet was resuspended in ATL buffer, and DNA was extracted using the Qiagen Inc. DNeasy PowerSoil Kit (Hilden, Germany). The amplicons were cleaned, indexed, and sequenced using the Illumina MiSeq platform. “Universal” 16S sequencing primers were utilized from the Earth Microbiome Project 16S Illumina Amplicon Protocol [35]. Forward 515F (5’-GTGYCAGCMGCCGCGGTAA-3’) [36] and reverse primers 806R (5’-GGACTACNVGGGTWTCTAAT-3’) [37] amplified the V4 hypervariable region.

### Amplicon Sequence Variant (ASV) determination and rarefication

A total of 15 gut samples and 17 nasal samples were provided for sequencing. ASVs were determined using the Divisive Amplicon Denoising Algorithm (DADA2; *version 1*.*16*.*1*) pipeline [38] in R (*Joy In Playing*; version 3.5.0). The forward primer and reverse-reverse complement primer were trimmed from the forward reads, and the reverse and forward-reverse complement were trimmed from the reverse reads. Forward reads were trimmed at 250 basepairs and reverse reads were trimmed at 200 basepairs after examining quality scores. Convergent error model parameterization and sample inference was estimated for forward and reverse reads [38]. Paired ends were merged, and chimeras were excluded [38]. Any sequence that was not taxonomically annotated as Bacteria or Archaea was excluded from further analyses. Collector’s curves were used to determine appropriate rarefaction depth. Because the number of unique ASVs per sample were asymptotic at the minimum read depth, nasal and gut samples were rarified without replacement (*set*.*seed(77)*) to the sample with the lowest sequencing depth (41,081 and 36,494 reads respectively). Rarefaction resulted in final ASV tables with 949 unique gut ASVs and 989 unique nasal ASVs.

### Determination of overall microbial community structure

Alpha diversity was calculated using the observed species richness, Shannon’s diversity index, and the phylogenetic alpha diversity with the *estimate_richness()* function of the phyloseq R package (version 1.34.0). Significant differences between alpha diversity measures in cats with and without clinical signs were evaluated using the Wilcoxon Rank Sum test.

To calculate beta diversity, microbial abundances were first transformed with the centered log-ratio (CLR) with the *transform()* function of the microbiome R package (version 1.12.0), and a redundancy analysis (RDA) plot was derived from the unweighted unique fraction metric (UniFrac) constrained on host status with the phyloseq R package (*ordinate(distance = “unifrac”)*). The vegan R package (version 2.5.7) *adonis()* function was used to conduct permutational multivariate analysis of variance (PERMANOVA) with and without controlling for household.

Taxa sums were calculated across all individuals, and the top six most abundant taxa at the phylum level for both gut and nasal microbiomes were subsetted for further analysis. Differential abundance between cats with and without clinical signs was assessed with the Wilcoxon Rank Sum tests for each phylum. After calculation, the resultant p-values were corrected for false discovery rate using the Bonferroni method.

### Determination of Cladal Taxonomic Units (CTUs)

Analyses were conducted separately for gut and nasal communities. ASVs were combined with full length reference 16S rDNA sequences from the All Species Living Tree Project (SILVA version 1.32) [39]. ASVs and reference sequences were aligned using mothur [40] (version 1.45.0). ASVs which did not align were disregarded from further analysis. A generalizable time-reversible phylogenetic model was constructed from the combined SILVA full length and ASV sequences using FastTree (*-gtr -nt*) (version 2.1.10). The resultant tree was midpoint rooted using the *midpoint()* function from the phangorn R package (version 2.7.1), and the reference sequences were subsequently pruned from the resultant phylogenetic tree. CTUs were determined by using the Cladal Taxonomic Unit (ClaaTU) algorithm as described previously [31]. In brief, ClaaTU conducts a root-to-tip traversal of the phylogenetic tree and quantifies the abundance of every monophyletic lineage within the reference tree within each sample. Taxonomy of each clade was assigned by determining the most specific Linnaean taxonomic label that was shared by all cladal descendants.

### Determination of microbial features associated with clinical signs

To determine microbial features (i.e. ASVs or CTUs) which were predicted by the presence or absence of clinical signs, we modeled microbial features as a function of host status using the negative binomial distribution with the *nb*.*glm()* function from the MASS package (version 7.3.54). If the model failed to converge, we did not include it in the final results. We considered only microbial features which occurred in at least >40% of individuals for models. To correct for multivariate hypothesis testing, a false discovery rate correction (*q* < *0*.*05*) was applied (*p*.*adjust(method = “fdr”)*). Features were visualized on the phylogenetic tree using the package ggtree (version 3.1.2.992).

### Determination of phylogenetic clustering or overdispersion of significant nodes

In order to determine if microbial features (ASVs and CTUs) phylogenetically clustered or were subject to overdispersion, we compared the phylogenetic distribution of selected features to null expectation using a bootstrapping approach. Specifically, we evaluated how the average pairwise phylogenetic distance of selected microbial features differs from the corresponding distance obtained from random subsamples of features across the 16S phylogeny. We also directly compared the distributions of the pairwise phylogenetic distances among selected features to the corresponding distance among randomly subsampled features from across the 16S phylogeny. In particular, we conducted these analyses on four different selected groups of features: (A) gut microbiome features positively associated with disease, (B) gut microbiome features negatively associated with disease, (C) nasal microbiome features positively associated with disease, and (D) nasal microbiome features negatively associated with disease.

For each group of selected features (A–D), we quantified the phylogenetic distance spanning all possible pairs of features using the *dist*.*nodes()* function in the *ape* R package (version 5.5). In order to simulate null expectation of pairwise phylogenetic distances, we randomly sampled *m* features from the 16S phylogeny, where *m*_*i*_ represents the number of significant features in each of the *i* selected feature groups {A, B, C, D}. We repeated this subsampling process 1,000 times for each selected feature set, and for each random subsample, quantified the phylogenetic distance spanning all possible pairs of the randomly subsampled nodes. In short, we produced size-matched, random feature sets for each group of selected features, so that we could determine if the phylogenetic characteristics of selected features significantly differed from the corresponding characteristics of random features, which would indicate phylogenetic clustering or overdispersion.

To determine if a selected group of features are on average more clustered or overdispersed than expected by chance, we quantified the average pairwise phylogenetic distance amongst the sets of features for each of the four selected feature groups. We also quantified the corresponding mean for each of the random subsets we produced for each group of selected features. Doing so produced a vector *r* of length 1,000 for each of the random subsamplings, where each element in the vector represented the average pairwise distances from a random subset of features. To determine if a given selected feature set’s observed average pairwise phylogenetic distance differed from the corresponding distance expected from randomly sampling the phylogeny, we conducted a z-test. Specifically, we used the formula below:

$$z_{i}=\frac{\mu_{i}-\mu (r_{i})}{\sigma (r_{i})}$$

where *μ*_*i*_ is the observed mean pairwise distance for each selected feature set, *μ*(*r*_*i*_*)* is the corresponding average of the expected mean pairwise distances, and *σ(r*_*i*_*)* is the standard deviation of these expected mean pairwise distances. A significantly small *z*_*i*_ indicates that the observed pairwise distances, are on average, less than expected by chance, indicating the selected features are consistent with phylogenetic clustering. Conversely, a larger *z*_*i*_ than expected by chance is consistent with phylogenetic overdispersion of the selected features.

Next, to account for the possibility that the point statistics (e.g., mean pairwise phylogenetic distance) may obscure patterns of phylogenetic variance that manifest across the entire dataset, we also evaluated the overall distribution of the phylogenetic distances between all pairs of selected features and compared this to a corresponding distribution of phylogenetic distances obtained from our bootstrapped data. To determine if a given selected feature set’s observed pairwise phylogenetic distance distribution differed from the corresponding distance distribution we would have expected from randomly sampling the phylogeny, we conducted a Kolmogorov-Smirnov (KS) test. Specifically, we used the formula below:

$$D=ks.test({dist}_{i},{dist\_rand}_{i})$$

were *dist*_*i*_ is the distribution of pairwise distances in the selected feature set and *dist_rand*_*i*_ is the corresponding size-matched pairwise distribution generated by random sampling of phylogenetic distances on the 16S phylogeny. Here, *D* stands for the KS distance statistic, which allowed us to test whether the selected feature set pairwise distance distribution was significantly different from a reference distance distribution. In this case, the reference distribution was obtained by randomly subsampling pairwise distances within the 16S phylogeny. This was repeated a total of 1,000 times, and each time, the *D-statistic* was recorded.

In order to determine if the observed vector of *D* test statistics differed from a corresponding set of *D* statistics that we would expect from only randomly sampling the phylogeny, we created a corresponding set of *D* statistics, *D’*, by comparing pairs of random subsets of pairwise distances using the KS test following the formula:

$$D\prime =ks.test({distance\_rand}_{i},{distance\_rand}_{i^{\prime }})$$

where *distance_rand*_*i*_ is the corresponding size-matched pairwise distribution generated by random sampling of phylogenetic distances on the 16S phylogeny. This was repeated a total of 1,000 times by selecting random pairs of bootstrapped datasets which allowed us to ensure that the number of comparisons among the random data is consistent with the number of comparisons that include the observed data. Finally, a Wilcoxon Rank Sum test was used to determine if the means of observed D test-statistics and expected D’ test-statistics significantly differed between the two populations. A significantly different mean D test-statistic indicates that the pairwise phylogenetic distances among selected features are generally distributed in a manner significantly different than that which would be expected by chance.

## Results

### Study design and participant characteristics

Cats were recruited based on a clinical history of the presence of chronic clinical signs consistent with infectious FURTD of greater than 1 year duration from 8 different households (Table 1). When available, cohabitating clinically normal cats were also sampled. Cats ranged in age from 4–14 years with a mean age of 7.4 years. Age was not statistically significant between FURTD and control groups (*t*.*test; p* = 0.3). There were 10 male-neutered and 7 female-spayed cats. The most common clinical signs of cats in the FURTD group reported by owners were nasal discharge, sneezing, ocular discharge, and ocular crusts. PCR testing for primary FURTD pathogens were positive in four of the cases and zero of the control individuals. For the final analyses, nasal microbiomes were sampled in 17 individuals (11 FURTD, 6 controls), and gut microbiomes were sampled in 15 individuals (10 FURTD, 5 controls) after exclusion criteria.

**Table 1:** Study population summary.

*ID*	Household	Age	Sex	Ocular	Nasal	Oral	Resp	Status	MB	PCR Result
*1*	A	9	MN	1	1	0	0	FURTD	N	Negative
*2*	A	4	MN	0	0	0	0	Control	G, N	Negative
*3*	B	11	FS	0	1	0	0	FURTD	G, N	FeHV-1
*4*	B	11	MN	0	0	0	0	Control	G, N	Negative
*5*	C	4	MN	0	0	0	0	Control	N	Negative
*6*	C	10	FS	1	0	0	0	FURTD	G, N	Negative
7*	D	6	MN	1	1	1	1	FURTD	N*	Negative
*8*	D	9	MN	1	1	1	0	FURTD	G, N	FCV, FeHV-1
*9*	D	6	FS	1	0	0	1	FURTD	G, N	*Bb*, FeHV-1, *Mf*
*10*	D	9	MN	1	0	1	0	FURTD	G, N	FCV
*11*	E	5	FS	1	1	0	1	FURTD	G, N	Negative
*12*	E	5	MN	0	0	0	0	Control	G, N	Negative
*13*	F	4	FS	1	0	0	0	FURTD	G, N	Negative
*14*	F	6	MN	0	0	0	0	Control	G, N	Negative
*15*	G	14	MN	1	0	0	0	FURTD	G, N	Negative
*16*	G	4	MN	1	1	0	0	FURTD	G, N	Negative
*17*	H	8	FS	1	0	0	0	FURTD	G, N	Negative
*18*	H	8	FS	0	0	0	0	Control	G, N	Negative

Participant characteristics. *ID*: Cat ID number. *Age*: in years. *Sex*: FS = Female Spayed; MN = Male Neutered. *Ocular/Nasal/Oral/Resp*: Signs present consistent with FURTD and confirmed by veterinary physical exam are encoded as 1 if present and 0 if otherwise. *Status*: Cats were classified into the FURTD group if they had one clinical sign consistent with infectious FURTD, and control otherwise. *MB*: N = samples collected for nasal microbiome sequencing. G = samples collected for gut microbiome sequencing. * = sample excluded based on exclusion criteria. *PCR Result*: FeHV-1 = Feline Herpes Virus Type 1, FCV = Calicivirus, *Bb* = *Bordetella bronchiseptica*, *Mf* = *Mycoplasma felis*.

### Markers suggestive of underlying inflammation differ in cats with and without clinical signs of chronic FURTD

**Fig 1:**
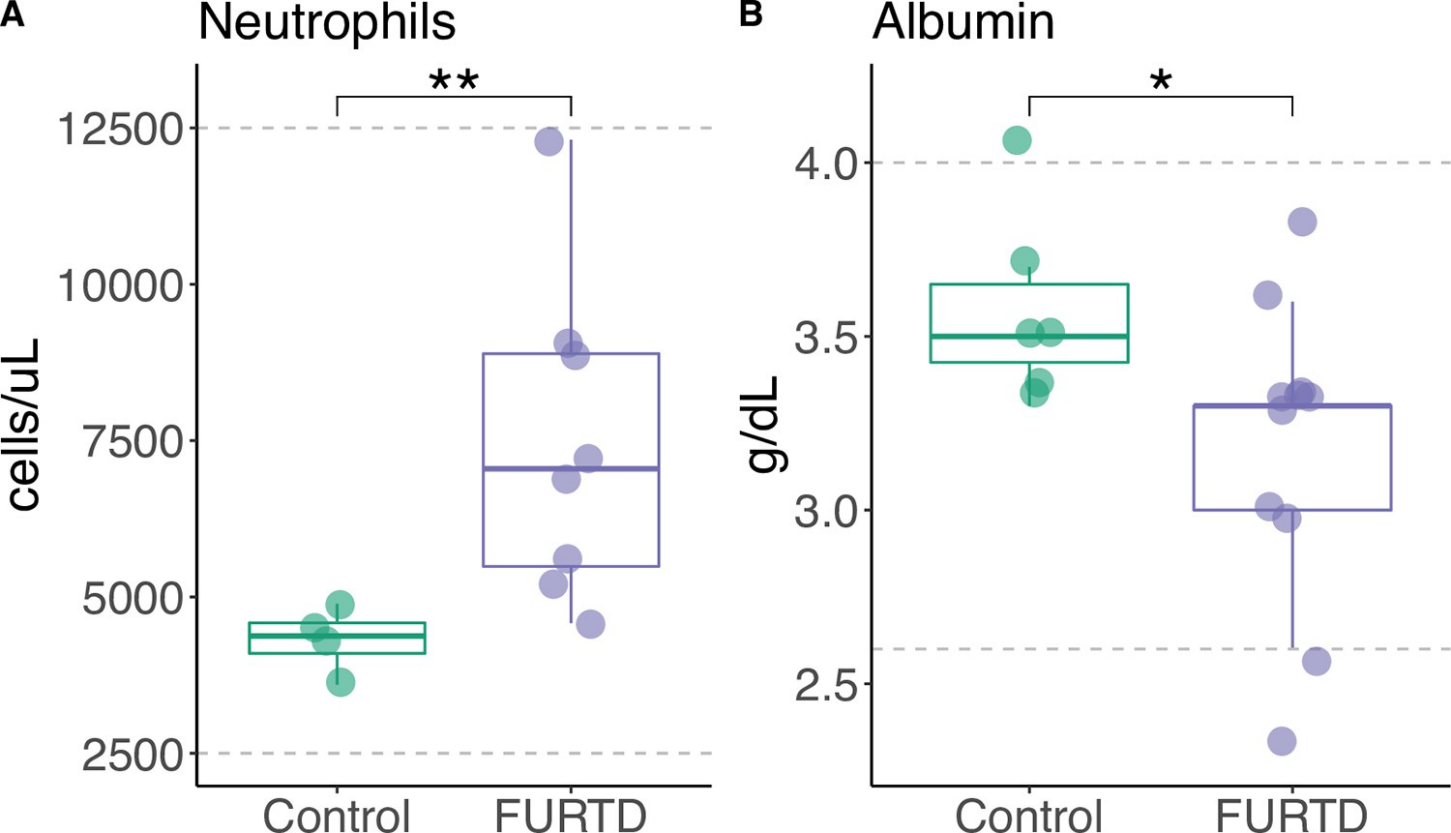
Markers suggestive of inflammation correlate with FURTD status. Absolute number of neutrophils (1A) and albumin levels (1B) are displayed for FURTD and control individuals. Laboratory reference ranges are indicated by grey dashed lines. Significance between groups is indicated by asterisks.

We hypothesized that markers suggestive of inflammation would differ in cats with and without clinical signs of FURTD due to systemic inflammatory effects of disease [40]. Although cell counts were within normal reference ranges for all cats, the number of neutrophils per microliter of whole blood was significantly increased in the FURTD group compared to controls (Fig 1A), consistent with an inflammatory response in the FURTD group (*Wilcoxon Rank Sum Test*; *W* = 31; *p = 0*.*01*). We also found that albumin levels were significantly lower in FURTD cats compared to controls (*W* = 11; *p = 0*.*03*), consistent with a negative acute phase protein response (Fig 1B). No significant differences were found between FURTD groups and controls for other blood values commonly associated with acute inflammatory responses such as elevations in globulins, immature (i.e. banded) neutrophils, and other leukocytes.

### Overall gut and nasal microbial community structure does not significantly associate with the presence of clinical signs

The most common microbial phyla present within the feline gut included Firmicutes, Bacteroidetes, Proteobacteria, and Fusobacteria (S1A Fig). Gut beta diversity did not significantly associate with the presence of clinical signs before (*Unifrac*; *PERMANOVA*; *R2* = 0.07; *p* = 0.40) or after (*PERMANOVA*; *R2* = 0.07; *p* = 0.19) controlling for household effects. Beta dispersion of gut microbial communities between cats with and without clinical signs was not significantly different (*F* = 0.56; *p* = 0.50).

Alpha diversity measures showed a trend towards higher diversity values in cats without clinical signs (S1A Fig), but this observation was not statistically significant (Wilcoxon Rank Sum; observed species richness, Shannon’s diversity index, and phylogenetic alpha diversity; *p* > 0.05). Only the relative abundance of Bacteroidetes was significantly less in the FURTD group compared to controls before false discovery rate (FDR) correction (*Wilcoxon Rank Sum*; *W* = 6; *p* = 0.023), however, there were no significant differences in gut microbiome taxonomic composition at the phyla level after FDR correction (Bonferroni; *q* > 0.05).

The most common microbial phyla present within the feline nasal microbiome included Proteobacteria, Bacteroidetes, Firmicutes and Fusobacteria (S1B Fig). Nasal beta diversity did not significantly associate with the presence of clinical signs before (*Unifrac*; *PERMANOVA*; *R2* = 0.06; *p* = 0.48) or after (*PERMANOVA*; *R2* = 0.06; *p* = 0.38) controlling for household effects. Beta dispersion of nasal microbial communities between cats with and without clinical signs was not significant (*F* = 2.24; *p* = 0.14).

Alpha diversity (S1B Fig) present within the cats with clinical signs and without clinical signs was not significantly different for all measures of alpha diversity tested (observed species richness, Shannon’s diversity index, and phylogenetic alpha diversity; *p* > 0.05). Relative abundances between community composition at the phylum level was not significant for the six most common phyla (*Wilcoxon Rank Sum test*; *p* > 0.05; Bonferroni; *q* > 0.05).

### Gut and nasal microbiome features significantly associate with presence of clinical signs

Next, we wished to explore if there were gut and nasal *microbial features* that associated with the presence of clinical signs. For the purposes of results and discussion, we refer to microbial features as the total combined set of ASVs (i.e., “microbial species”) and CTUs (i.e., monophyletic microbial lineages). Using a recently developed ecophylogenetic framework [31, 41], we computed how each CTU distributed across samples using a phylogenetic tree constructed from ASVs and the microbial community distribution. Incorporation of phylogeny into the assessment of how microbial lineages distribute across samples allows for determination of monophyletic clades of microbes that collectively manifest patterns based on host disease status that might not otherwise be noted using taxonomic labels alone. For example, the limited and punctuated resolution of pre-defined taxonomic labels, which are often used for microbiome analyses, can obscure signals present at more or less granular resolution. Identification of clades linked to host status has the added benefit to resolve putative trait origins that underly such observed associations.

Overall, there were a total of 1,897 gut microbial features, of which 136 (7.2%) were significantly associated with the presence of chronic FURTD signs after correction for multiple hypothesis testing (*q < 0*.*05*) (S2 Fig). Taxonomic labels of gut microbial features most commonly associating with control individuals included Lachnospiraceae (*n* = 6), Porphyromonadaceae (*n* = 5), Prevotellaceae (*n* = 5), and Ruminococcaceae (*n* = 5). Gut microbial families most commonly associating with cats with chronic FURTD signs included Lachnospiraceae (*n* = 15), Bacteroidaceae (*n* = 13), and Clostridiales Family XI (*n* = 11). It is important to note that, if analysis was carried out using taxonomic labels alone, it is possible that some of the groups reported above would not have been discovered. For example, multiple clades of Lachnospiraceae were identified for both FURTD and control individuals. Had these descending lineages been conglomerated into a single taxonomic label, the conflicting association between control and FURTD individuals would have likely mitigated any signal found.

Of the total 1,977 nasal microbiome features, only 89 (4.5%) were significantly associated with the presence of chronic FURTD signs (*q < 0*.*05*) (S3 Fig). The family-level taxonomic labels most commonly associated with control individuals were Porphyromonadaceae (*n* = 7), Peptostreptococcaceae (*n* = 4), Pasteurellaceae (*n* = 4), Prevotellaceae (*n* = 3), Clostridiales Family XII (*n* = 3), and Spirachaetaceae (*n = 3*). Microbial feature family-level taxonomic labels most commonly associated with FURTD individuals included Spirochaetaceae (*n* = 6), Actinomycetaceae (*n* = 4), Porphyromonadaceae (*n* = 4), Pasteurellaceae (*n = 4*), and Moraxellaceae (*n* = 4).

### Gut microbiome lineages show conserved patterns of association with host disease status

Of the 136 gut microbial features that had significant associations with the host, there were 31 individual ASVs and 105 CTUs which showed patterns of association within the gut microbiome. We hypothesized that descendants within these microbial lineages may show similar association patterns with host status. We reasoned that related groups of microbes are more likely to have more related functional capacity, and thus are more likely to share similar patterns of association with the host. Because of this, we hypothesized that there would be positions on the phylogenetic tree that showed phylogenetic clustering (i.e., “hot spots” of association) with the host status.

We found that microbial features of the gut significantly associating with host clinical signs exhibited evidence of phylogenetic clustering. In order to evaluate the presence of phylogenetic clustering, we implemented a bootstrapping method for determining the null expectation of (A) pairwise *distances* between randomly chosen nodes on the microbial phylogenetic tree, as well as (B) pairwise *distance distributions* of random nodes. We then compared the null expectation to the matching set of statistics generated from the selected microbial features that either positively or negatively associated host status.

**Fig 2:**
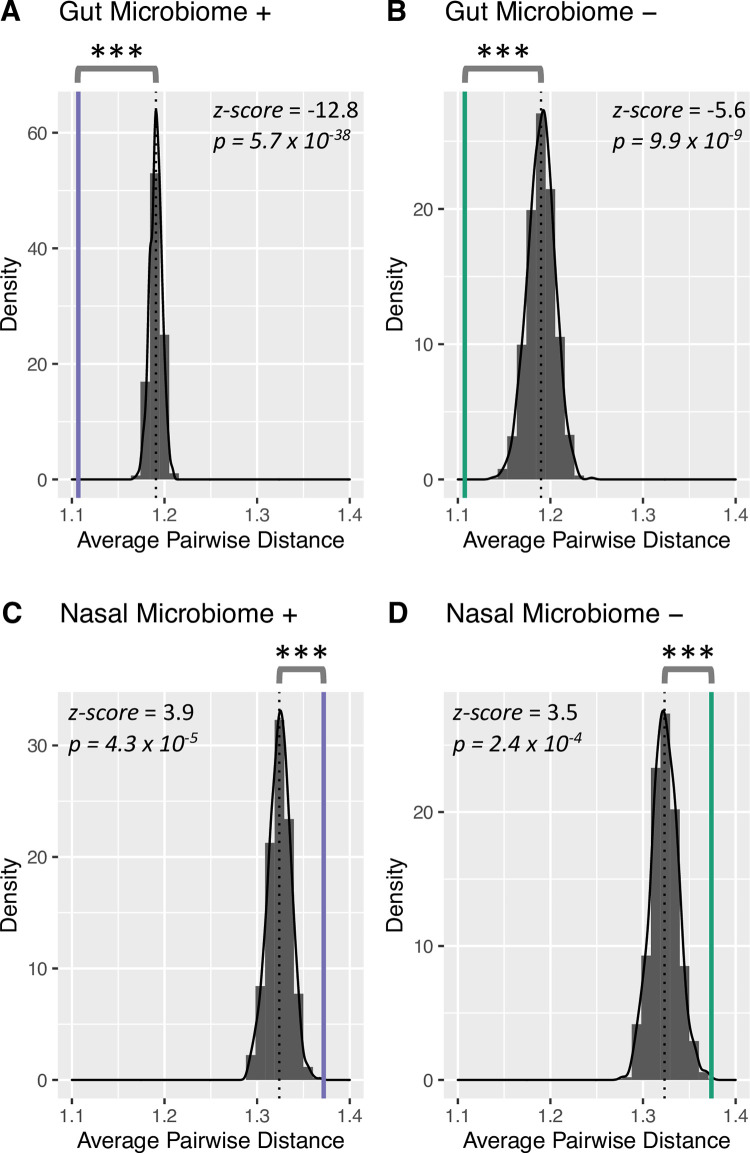
Gut microbiome features significantly associated with clinical signs show evidence of phylogenetic clustering while nasal microbiome features show evidence of phylogenetic overdispersion. Microbial features that were significantly associated with clinical signs were separated into four groups: gut microbiome features that positively (A) or negatively (B) associated with clinical signs, and nasal microbiome features that positively (C) or negatively (D) associated with clinical signs. For each figure, the observed average pairwise distance is indicated by a vertical line. The null expectation of the average pairwise distance between two random nodes observed on the phylogenetic tree is indicated by the black dotted vertical line. The null expectation average pairwise distance was generated from a bootstrapped set of random samplings of phylogenetic distances (*N =* 1,000), and is indicated by the gray bars. The corresponding *z-score* calculated from the selected feature set compared to the corresponding null expectation is indicated in each figure. Significance is indicated by asterisks.

First, to determine the significance of pairwise *distances* of microbial features that associated with clinical signs, we calculated a *z-score* of the observed pairwise distances of significant gut microbiome features and compared them to a bootstrapped null expectation of average random pairwise distances on the gut microbial tree (*N* = 1,000*)*. We found that the average pairwise distance (*μ_gut_*) for gut microbial features that positively (*μ*_*gut*+_ = 1.11; *σ*_*gut*+_ = 0.43) and negatively (*μ*_*gut*−_ = 1.11, *σ*_*gut*−_ = 0.43) associated with clinical signs was significantly less than null expectation (Fig 2A and 2B) (*z-score*; *p* << 0.001).

**Fig 3:**
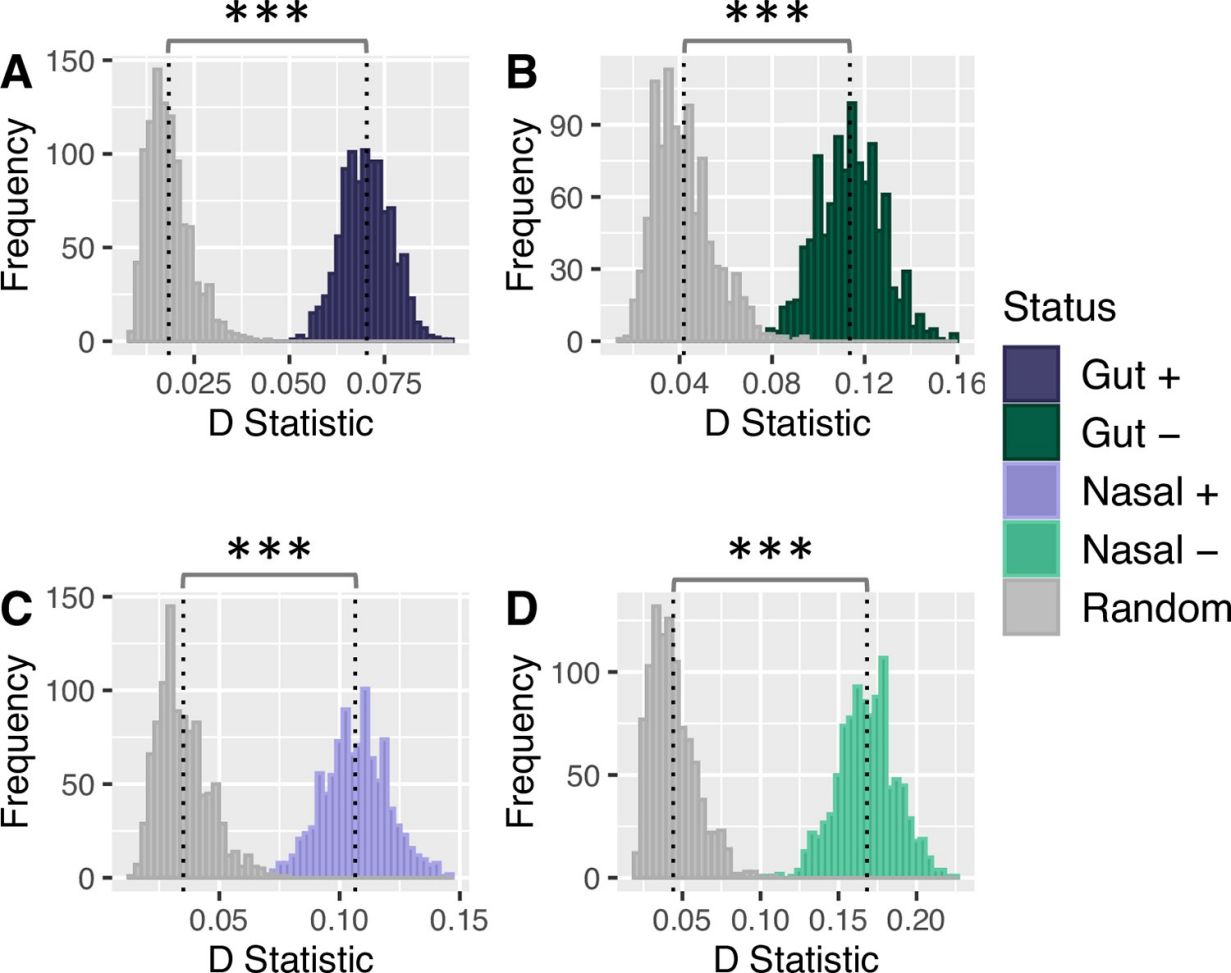
Gut and nasal microbiome features have significantly different pairwise distance distributions compared to null expectation. Microbial features that were significantly associated with clinical signs were separated into four groups A–D. For each group, the observed *D* statistic was calculated by comparing the observed pairwise distances against a random sampling of nodes using the KS test and is indicated by colored bars. A corresponding null set of *D* statistics, *D’*, was generated by comparing pairwise distance distributions from two random sampling events on the phylogenetic tree and is indicated by grey bars. This was repeated for a total of 1,000 times for each case. The mean *D* and *D’* statistic value is indicated by black dotted lines. Statistical significance between groups was assessed using the Wilcoxon Rank Sum Test. Mean *D* statistic for all selected features was significantly greater compared to null expectation (*p* < 2.2 x 10^−16^).

Second, to account for the possibility that point statistics (e.g., mean distance) may obscure patterns that manifest across the entire data set, we also evaluated the overall distribution of phylogenetic distances between all pairs of significant features by calculating a *D statistic* (see methods). We then compared the distribution of *D* statistics of selected features to a corresponding null distribution obtained from our bootstrapped data. We found that the mean *D* statistic value was significantly higher amongst the microbial features that both positively and negatively associated with the presence of clinical signs compared to null expectation (*Wilcoxon Rank Sum Test*; *p << 0*.*001*) (Fig 3A and 3B). Taken together, these results indicate that there is evidence of phylogenetic clustering of gut microbiome features that significantly associate with clinical signs.

**Fig 4:**
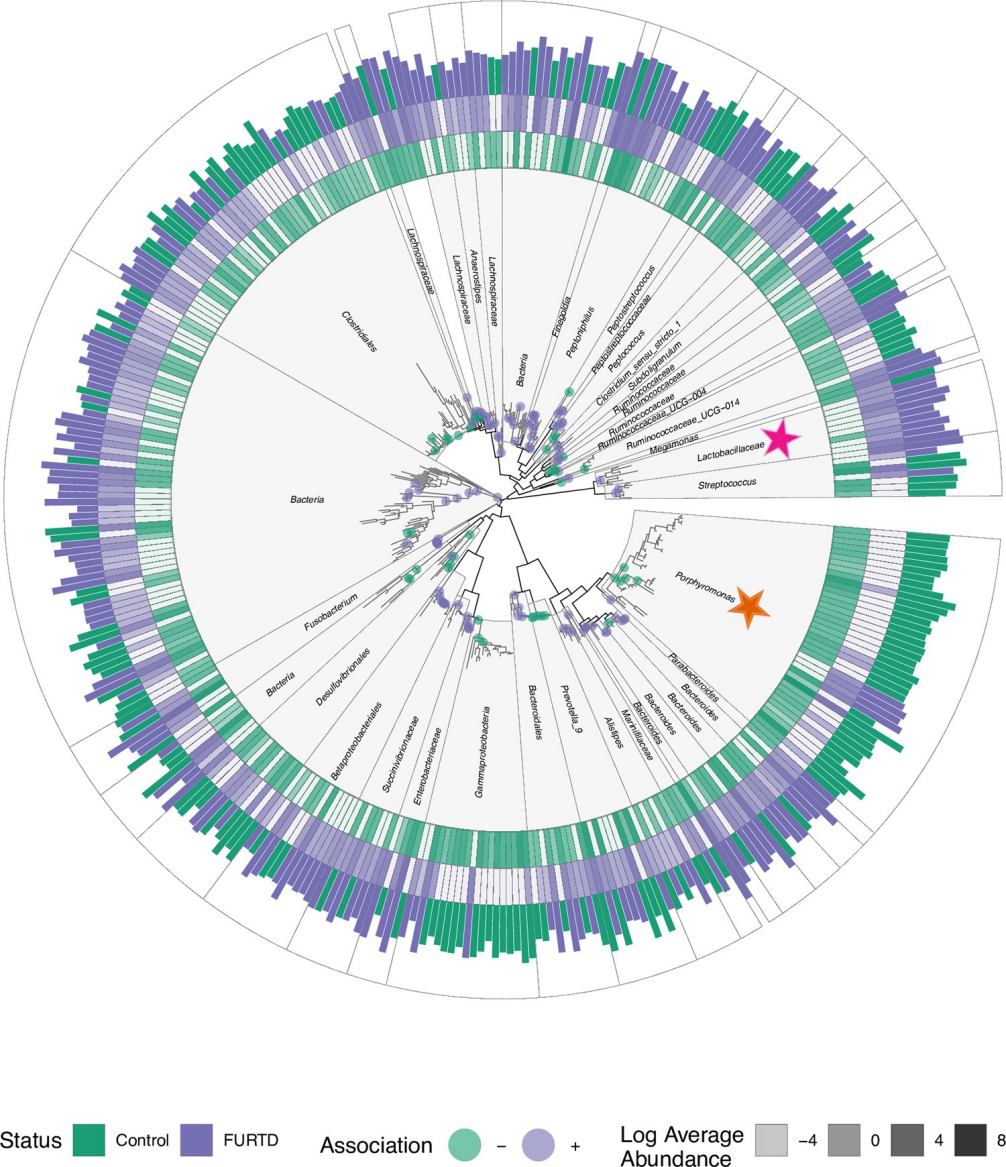
Monophyletic lineages within the gut microbiome reveal distinct cladal patterns of association with clinical signs. CTUs and ASVs significantly associating with clinical signs were subset and displayed. Clade taxonomic labels were assigned by determining the most specific Linnaean taxonomic label that was shared by all cladal descendants. Significant associations with FURTD or control individuals indicated with purple and green points respectively. Tiles represent the log transformed average abundances of each ASV in individuals without (*green tiles*, *inner ring*) and with (*purple tiles*, *middle ring*) clinical signs. Bar colors along the outside ring of the figure represent the average abundance of the status group with the higher value for that particular ASV. For example, a bar is colored green if cats within the control group had a higher average abundance for that particular ASV compared to FURTD cats. In this example, the bar size would correspond to the average ASV abundance for cats within the control group. Orange and pink stars are referred to as points of interest in the text.

Examining the association of each descendant within lineages significantly associated with host status, we found some striking examples of conservation of host microbe associations (Fig 4). For example, several related ASVs taxonomically annotated as Porphyromonas (Phyla Bacteroidetes; Order Bacteroidales) are all increased in control cats compared to FURTD individuals (orange star). Conversely, we found that in cats with clinical signs, there is a relative increased abundance of a particular clade taxonomically annotated as the family Lactobacillaceae (pink star). This lineage contained 9 different members, 8/9 of which were annotated as *Lactobacillus*, and 1/9 of which was annotated as *Pediococcus*.

### Nasal microbiome lineages show conserved patterns of association with host disease status

Of the 89 microbial features of the nasal microbiome that had significant associations with chronic FURTD signs, 45 were individual ASVs and 44 were CTUs. As before, we hypothesized that descendants within these microbial lineages may show similar association patterns with host status. We reasoned that related groups of microbes are more likely to have more related functional capacity, and thus are more likely to share similar patterns of association with the host. Because of this, we hypothesized that there would be positions on the phylogenetic tree that showed phylogenetic clustering (i.e., “hot spots” of association) with the host status.

In contrast to the gut microbiome and our expectations however, microbial features of the nasal microbiome significantly associating with clinical signs did not exhibit evidence of phylogenetic clustering but instead showed evidence of over-dispersion (Fig 2C and 2D). We found that for nasal microbial features both positively (*μ*_*nasal*+_ = 1.37; *σ*_*nasal*+_ = 0.40) or negatively (*μ*_*nasal*−_ = 1.37; *σ*_*nasal*−_ = 0.48) associating with clinical signs, that the average pairwise distance (*μ_nasal_*) was significantly greater than would be expected under null expectation (*z-score*; *p* << 0.001). Like the gut microbiome, we found that the distribution of pairwise node distances was significantly different compared to null expectation (Wilcoxon Rank Sum Test; *p << 0*.*01*) (Fig 3C and 3D).

**Fig 5:**
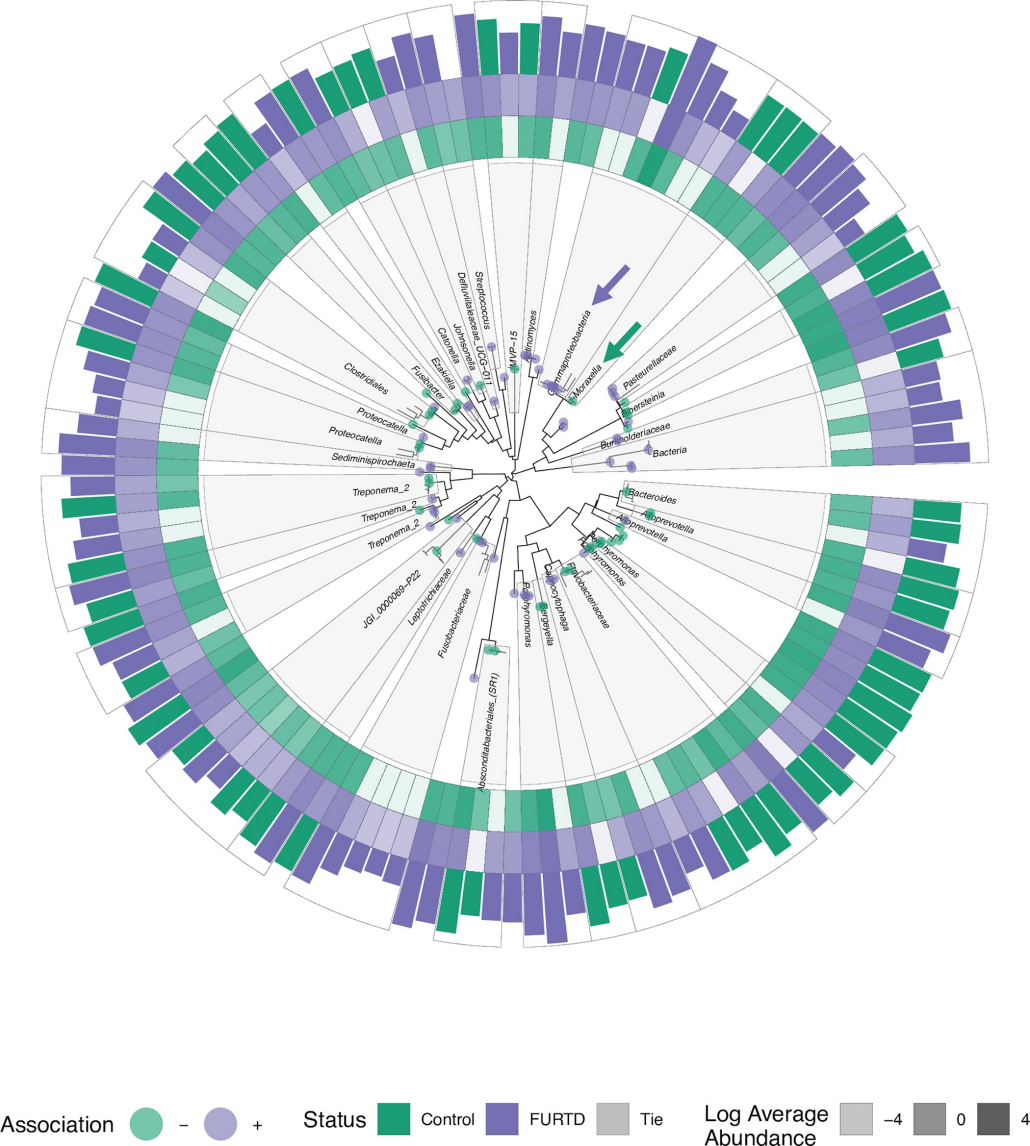
Monophyletic lineages within the nasal microbiome reveal distinct cladal patterns of association with clinical signs. Monophyletic lineages and ASVs within the nasal microbiome significantly associating with clinical signs were subset and visualized. Clade taxonomic labels were assigned by determining the most specific Linnaean taxonomic label that was shared by all cladal descendants. Significant microbial features with FURTD or control individuals are notated on the phylogenetic tree with purple and green points respectively. Tiles represent the log transformed average abundances of each ASV in individuals without (*green tiles*, *inner ring*) and with (*purple tiles*, *middle ring*) clinical signs. Bars colors along the outside ring represent the average abundance of the status group with the higher value for that particular ASV. For example, a bar is colored green if cats within the control group had a higher average abundance for that particular ASV compared to FURTD cats. In this example, the bar size would correspond to the average ASV abundance for cats within the control group. Purple and green arrows are referred to as points of interest within the text.

Cladal information reveals unique insight into patterns of association of nasal microbiome features. For example, consider the heterogeneity of association of clades with the taxonomic label within the Class Gammaproteobacteria and Family Moraxellaceae (Fig 5). One lineage taxonomically annotated as Moraxella has descending lineages which all show increased abundances in control cats (green arrow), while another clade within Gammaproteobacteria contains six descending lineages of *Moraxella* displaying association with presence of clinical signs (purple arrow).

## Discussion

Understanding the differences in community compositions between cats with and without chronic signs of FURTD may provide new insight into how the microbial community interacts with the primary pathogens leading to development of chronic clinical disease. We identified specific microbial features that linked with presence or absence of chronic clinical signs. Understanding the taxonomic community composition and functional capacity of the feline upper respiratory tract and gut microbiome and their respective roles in chronic FURTD pathogenesis may provide new lines of study that could produce novel therapeutics or prognostics for cats with infectious FURTD. Given our study design is associative in nature, it is impossible to determine if microbial communities significantly associating with chronic clinical course either (A) cause the presence of chronic clinical signs, or (B) result from the presence of clinical signs.

Regardless of the directionality of the interaction, we believe that our work provides a baseline for future clinically relevant studies. For example, if the gut microbiome is a causal mediator of clinical signs (case A), understanding which microbes can mitigate primary pathogen effects may lead to new probiotics that could lessen clinical signs of FURTD. If the directionality of the associative interaction identified here is reversed, and microbial member abundances result from presence of clinical signs (case B), microbial features identified that associate with diseased individuals may hold valuable prognostic information to predict persistent presence or recurrence of clinical course in cats. Future work in this line of inquiry could lead to development of diagnostics to aid in determining the risk of chronic clinical disease given the presence or absence of these high information “biomarker” microbial CTUs and ASVs. Regardless of the directionality of the associative interaction, the clades and individual microbial members identified here provide a baseline for prioritization of microbes with potential clinical impact, whether it be their ability to manipulate microbial communities to influence clinical course, or to identify the priority targets for providing the diagnostician with prognostic information regarding disease course.

In our study, we found evidence of phylogenetic clustering within significant gut microbial features associating with clinical signs. Phylogenetic clustering is consistent with a conserved phylogenetic core of clades that are present in cats with and without clinical signs compared to a random sampling of nodes. Clustered clades could represent a core set of related microbial lineages that either expand during the presence of clinical disease or that exist normally during health which become disrupted during disease.

In contrast, significant nasal microbiome features associating with clinical signs were overdispersed. Phylogenetic overdispersion of nasal microbial features associating with clinical signs is an interesting contrast from the phylogenetic clustering that was observed within the gut microbiome. Overdispersion may suggest that nasal microbes which significantly link to presence or absence of active clinical signs are opportunistic or competitively exclude related microbial members. Alternatively, overdispersion could suggest that microbial traits associating with clinical signs are phylogenetically overdispersed among microbial members or less subject to vertical evolution among microbes, as compared to those traits that link gut microbial lineages to clinical signs.

An overall inflammatory state provides a putative mechanism for restructuring gut and nasal microbial communities. As expected, we found that cats with chronic FURTD signs had results suggestive of increased levels of inflammation as indicated by significantly higher neutrophil counts and reduction in albumin, a negative acute phase protein. It is probable that during an inflammatory state, the host tends to filter for particular monophyletic lineages of microbes within the gut and nasal passages via conserved microbial traits. Our study also suggests that clinically relevant changes in absolute neutrophil number and albumin still exist within normally established reference ranges. It is important to note the two markers measured here (neutrophil count and albumin) are suggestive of underlying inflammatory state, though they do not confirm it. Future work should seek to include additional measures of inflammatory state such as positive acute phase proteins (e.g., C-reactive protein) along with performing PCR of various cytokine concentrations linked to inflammation (e.g., TNF-alpha).

Our findings link the gut microbiome to the presence of chronic FURTD signs. Microfold cells (M-cells) are cells that sample microbes within the gut and lay within the epithelia overlying the gut-associated lymphoid tissues (GALT) [42]. M-cells allow for transcytosis of luminal antigens to mononuclear phagocytes, thereby inducing mucosal immune responses such as IgA production [42]. Recently, cells with the typical features of M-cells have been found within murine nasal passage epitheliums, overlying a novel Nasal Associated Lymphoid Tissue (NALT). NALT enables independent sampling of pathogens within the respiratory tract [42]. It is well known that there is crosstalk between different mucosal systems within the body, so IgA production in the NALT may affect gut microbiomes and vice versa [43]. For example, many studies have shown cross mucosal interaction at distant sites, such as mucosal vaccination leading to protection at another mucosal surfaces, infection with a virus at one mucosal site resulting in IgA secretions at distal sites, and greater risk of respiratory allergies in neonates that are put on a course of antibiotics [43].

We discovered several microbial clades within the gut which are known to have probiotic properties mediating the gut—respiratory axis in higher abundances in cats with chronic signs compared to those without. This apparent paradox has been described previously in studies that monitor enteric gut microbiome population response upon infection with respiratory virus [44]. For example, we discovered a higher number of microbes taxonomically annotated as *Lactobacillus* in cats with signs of chronic FURTD. *Lactobacillus* has classically been associated with protection from respiratory viral infection in several disease models due to its established role as a probiotic mediator of the gut-lung axis. Changes within enteric microbial populations upon infection with respiratory virus is less studied, but prior studies speculate that host recruitment of microbes with anti-inflammatory properties may provide a host resilience to viral infection. For example, expansions of murine enteric populations of *Lactobacillus* have been shown to increase upon infection with influenza virus [44], and decreasing *Lactobacillus* numbers upon respiratory viral infection has been shown to increase risk of death in a mouse model [45]. The authors speculate that increases in gut *Lactobacillus* numbers may therefore be a host adaptive response to viral infection. Administration of *Lactobacillus* strains in mice can decrease asthmatic symptoms [46–49], decrease airway inflammation and alveolar damage [49, 50], and provide protection against respiratory viral infection [23, 45, 51]. Thus, we might expect *Lactobacillus* numbers to increase in those animals with chronic FURTD signs compared to controls as an adaptive response.

We found a relatively higher abundance of microbes within Porphyromonas within the order Bacteroidales in control individuals. Reduction in relative abundance of Bacteroidales has been described with respiratory viral infection in mouse models previously. For example, there was a dramatic reduction of gut Bacteroidales numbers upon infection with influenza A virus [44], but the authors did not further describe if all members of Bacteroidales taxa exhibited similar reductions or if some members within the taxonomic group exhibited opposite effects. In our study, reduction of Bacteroidales was driven largely by a diffuse association with subtending lineages of the Porphyromonas. Other members within the Bacteroidales family did not manifest similar patterns of association with clinical signs.

We found several lineages within the nasal microbiome that were linked to the presence of chronic FURTD course. One such lineage labeled as the class Gammaproteobacteria had opposite associations with host status–one group of descending lineages (labeled as *Moraxella*) had negative associations with host disease status, while other descending lineages (also labeled as *Moraxella*) within the same class positively associated with clinical signs. *Moraxella* isolates have been noted for their heterogeneity in ability to cause clinical signs previously [52]. While some lineages of *Moraxella* are commensals, others are considered important respiratory pathogens that can worsen other diseases of the respiratory tract such as chronic obstructive pulmonary disease [53]. In humans, recent acquisition of one of two distinct lineages of *Moraxella catarrhalis* has been linked to exacerbations of airway inflammation and increased clinical signs [53–55]. Some *Moraxella* strains in humans produce β-lactamase, and so are resistant to ampicillin. In addition, *Moraxella* resists antibiotic treatment by its ability to form biofilms, recently implicated in recurrent and chronic otitis media and upper respiratory symptoms in children [56]. Another clade which is strongly linked to presence of FURTD clinical signs is *Fusobacterium*, which is also one of the most common isolates in humans with chronic sinusitis [57].

Our ecophylogenetic approach yields unique discoveries that examination of taxonomic labels or ASVs alone cannot. Considering each and every CTU’s association with host status can reveal divergent functional groups. For example, consider if the clade *Moraxella* had been collapsed at genus level to a single label and then assessed for association with host status. Because some descendants have a positive association with disease and others have a negative association with disease, if we were to consider the group as a whole, it is possible that neither would have been significantly associated with host status. Also, an ecophylogenetic approach yields groups of descendants that likely have conserved traits of association with host disease status that could be followed up on with comparative genomics approaches to identify specific bacterial mechanisms of interaction with host.

Our work suffers from several limitations. First, while our study benefits from being able to rapidly determine microbial features that associate with FURTD clinical signs, it is limited by its cross-sectional nature. Because of this, the directionality of cause and effect cannot be determined. It is possible that the microbes found in higher abundances in FURTD cats contribute to the severity of clinical signs. Conversely, it is possible that clinical signs lead to selection of different microbial communities. Second, we are limited by the relatively small number of cats present in our study. Future work should seek to recruit larger cohorts in order to identify the robustness of associations determined here. Third, our study is limited by the fact that many different infectious agents can lead to chronic clinical course observed in infectious FURTD. It is possible that each primary pathogen associates with unique changes in the gut and respiratory microbiomes. Future work should seek to unravel the specific association of each primary pathogen with the gut and nasal microbiomes. Fourth, our work is limited by the lack of definitive diagnosis for the cats in question. It is possible that there were other causes besides infectious FURTD leading to chronic nasal signs within our recruited population. Additional studies should seek to definitively rule out the presence of other diseases leading to chronic upper respiratory tract signs through use of additional diagnostics (e.g., biopsy and rhinoscopy). Unfortunately, due to cost constrains and the invasive nature of rhinoscopy/biopsy, use of these tests was not feasible in the current study. Last, we only considered the bacterial component of the nasal and gut microbiomes in regards to FURTD. Future work should seek to characterize fungal communities within nasal passages given that fungal organisms such as *Cryptococcus* can be a cause of infectious FURTD. It is also possible that other pathogens contribute to the disease process that have yet to be described. Future work should consider virome as well as microbiome sequencing to identify other possibilities of host-viral interactions that lead to long term disease sequalae.

The gut and nasal microbiome may be a useful prognostic indicator of chronic clinical disease upon acute infection. Future work should seek to determine the reason some cats develop chronic clinical disease and some do not. For example, do the microbial lineages which were increased in control compared to FURTD cats have “protective” effects against development of chronic clinical course? Are microbes that are associated with chronic disease present upon initial infection with the primary pathogen, or do they establish after acute infection of the primary pathogen? We were not able to answer these questions because our work only considered one time point, but future studies which establish how microbial communities respond to initial infection and then develop during chronic clinical disease will shed light on cause and effect. Future work describing the interactions between the gut and respiratory microbiomes could provide the basis for novel therapeutics. Ultimately, protective probiotic strains given to shelter animals on intake may reduce the amount of morbidity associated with initial infection, as well as decrease the rate of chronic clinical disease.

## Supporting information

S1 Table Animal survey metadata and animal laboratory database. (XLSX)

S1 Fig Gut and nasal microbiome community overview. Gut (**A**) and nasal (**B**) microbiome communities. The top row of each panel contains an overview of alpha (right) and beta (left) diversity summaries. The bottom row contains the relative abundances of the six most common phyla broken down by host status. The presence (purple) or absence (green) of clinical signs (host status) is noted by color for both beta and alpha diversity plots. **A** *Beta Diversity*: Redundancy analysis (RDA) plot derived from unweighted unique fraction metric (UniFrac) constrained on host status (*Unifrac*; *PERMANOVA*; *R2* = 0.07; *p* = 0.40). The percentage of variance explained by each PC is displayed in descending order after the constrained axis (orange bar). Host status explained 7.6% of variation within gut microbiome community compositions. Beta dispersion was not significant between groups (*PERMDISP2*, *F* = 0.56; *p* = 0.50). *Alpha Diversity*: Observed species richness (*Wilcoxon Rank Sum*; *W* = 35; *p* = 0.25), Shannon’s diversity index (*Wilcoxon Rank Sum*; *W* = 33; *p* = 0.37), and phylogenetic alpha diversity (*Wilcoxon Rank Sum*; *W* = 38; *p* = 0.13) was not significant between groups. *Taxonomic Composition*: Wilcoxon Rank Sum tests were performed on relative abundances for each taxonomic grouping based on host status, and then the resultant p-values were corrected for false discovery rate (Bonferroni). No significant phyla level differences were observed between cats with and without clinical signs after correction for false discovery rate (*q* > 0.05): Firmicutes (*W* = 36; *p* = 0.20; *q* = 1.0), Bacteroidetes (*W* = 6; *p* = 0.023; *q* = 0.14); Proteobacteria (*W* = 34; *p* = 0.30; *q* = 1.0), Fusobacteria (*W* = 16; *p* = 0.30; *q* = 1); Actinobacteria (*W* = 40; *p* = 0.08; *q* = 0.45); Epsilonbacteraeota (*W* = 29; *p* = 0.67; *q* = 1.0). **B** *Beta Diversity*: RDA plot derived from unweighted UniFrac constrained on host status (*Unifrac*; *PERMANOVA*; *R2* = 0.06; *p* = 0.48). The percentage of variance explained by each PC is displayed in descending order after the constrained axis (orange bar). Host status explained 5.4% of variation within nasal microbiome community compositions. Beta dispersion was not significantly different between groups (*PERMDISP2*, *F* = 2.24; *p* = 0.14). *Alpha Diversity*: Observed species richness (*Wilcoxon Rank Sum*; *W* = 45; *p* = 0.27), Shannon’s diversity index (*Wilcoxon Rank Sum*; *W* = 31; *p* = 0.88), and phylogenetic alpha diversity (*Wilcoxon Rank Sum*; *W* = 36; *p* = 0.81) was not significant between groups. *Taxonomic Composition*: Wilcoxon Rank Sum tests were performed on relative abundances for each taxonomic grouping based on host status, and then the resultant p-values were corrected for false discovery rate (Bonferroni). No significant phyla level differences were observed between cats with and without clinical signs (*q* > 0.05): Proteobacteria (*W* = 26; *p* = 0.51; *q* = 1.0), Bacteroidetes (*W* = 27; *p* = 0.58; *q* = 1.0), Firmicutes (*W* = 40; *p* = 0.51; *q* = 1.0), Fusobacteria (*W* = 34; *p* = 0.96; *q* = 1.0), Spirochaetes (*W* = 42; *p* = 0.39; *q* = 1.0), and Patescibacteria (*W* = 40; *p* = 0.51; *q* = 1.0).
(TIF)

S2 Fig Gut microbiota features significantly associate with the presence of clinical signs. Each microbial feature was modeled as a function of clinical signs. Features of the gut microbiome which significantly associated with the presence (purple triangles) or absence (green circles) of clinical signs are indicated by tree node color (*q < 0*.05). Phylogenetic tree labels indicate the significant microbial feature’s phylum-level taxonomic label.
(TIF)

S3 Fig Nasal microbiota features significantly associate with the presence of clinical signs. Each microbial feature was modeled as a function of clinical signs. Features of the nasal microbiome which significantly associated with the presence (purple triangles) or absence (green circles) of clinical signs are indicated by tree node color (*q < 0*.*05*). Phylogenetic tree labels indicate the significant microbial feature’s phylum-level taxonomic label.
(TIF)
