# Supplementary figures and images for: Chronic clinical signs of upper respiratory tract disease associate with gut and respiratory microbiomes in a cohort of domestic felines
Source: PLoS One. 2022 Dec 1;17(12):e0268730. doi: 10.1371/journal.pone.0268730 (PMC9714858; doi:10.1371/journal.pone.0268730)

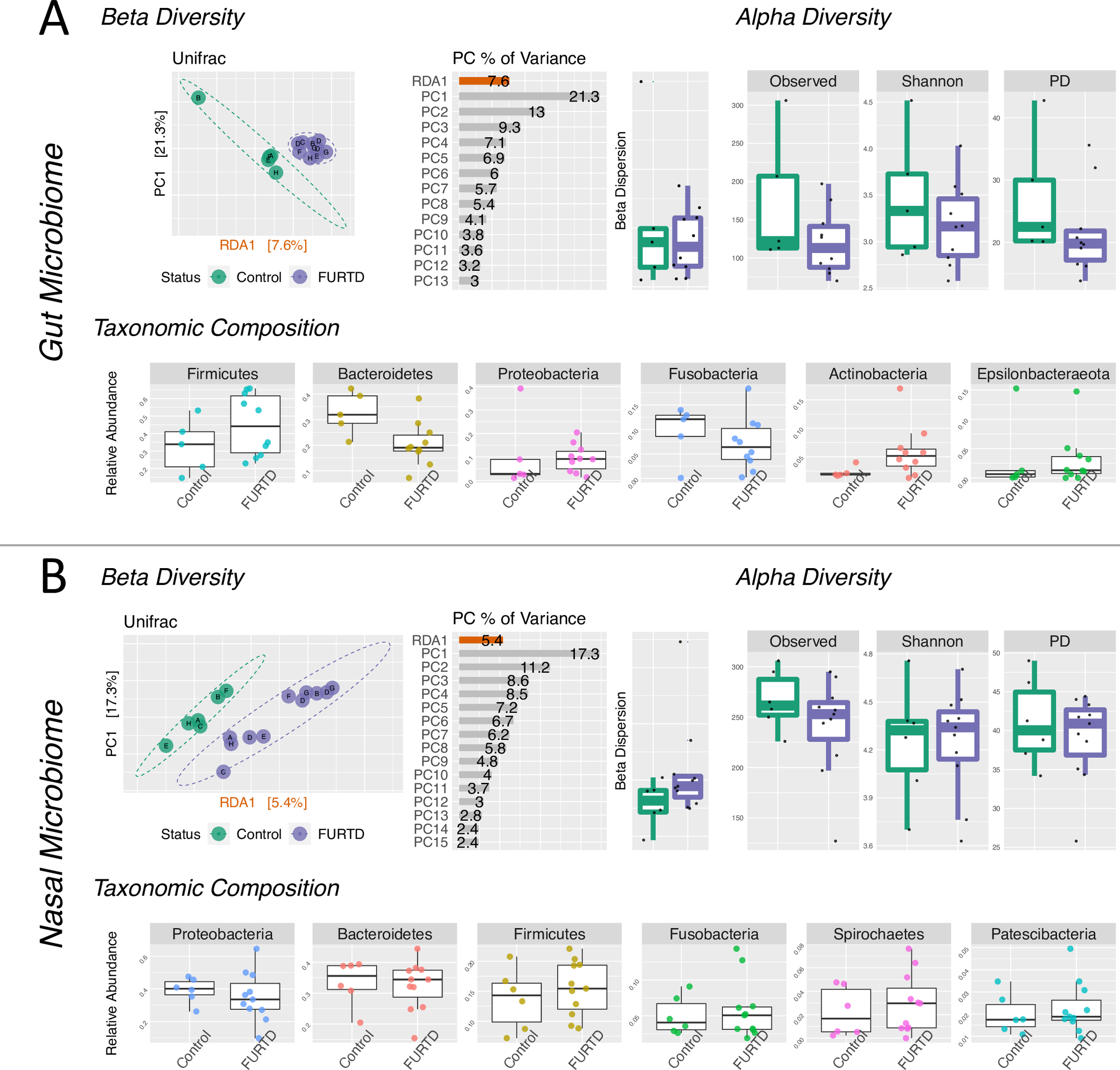

Supplement: S1 Fig — Gut (A) and nasal (B) microbiome communities. The top row of each panel contains an overview of alpha (right) and beta (left) diversity summaries. The bottom row contains the relative abundances of the six most common phyla broken down by host status. The presence (purple) or absence (green) of clinical signs (host status) is noted by color for both beta and alpha diversity plots. A Beta Diversity: Redundancy analysis (RDA) plot derived from unweighted unique fraction metric (UniFrac) constrained on host status (Unifrac; PERMANOVA; R2 = 0.07; p = 0.40). The percentage of variance explained by each PC is displayed in descending order after the constrained axis (orange bar). Host status explained 7.6% of variation within gut microbiome community compositions. Beta dispersion was not significant between groups (PERMDISP2, F = 0.56; p = 0.50). Alpha Diversity: Observed species richness (Wilcoxon Rank Sum; W = 35; p = 0.25), Shannon’s diversity index (Wilcoxon Rank Sum; W = 33; p = 0.37), and phylogenetic alpha diversity (Wilcoxon Rank Sum; W = 38; p = 0.13) was not significant between groups. Taxonomic Composition: Wilcoxon Rank Sum tests were performed on relative abundances for each taxonomic grouping based on host status, and then the resultant p-values were corrected for false discovery rate (Bonferroni). No significant phyla level differences were observed between cats with and without clinical signs after correction for false discovery rate (q > 0.05): Firmicutes (W = 36; p = 0.20; q = 1.0), Bacteroidetes (W = 6; p = 0.023; q = 0.14); Proteobacteria (W = 34; p = 0.30; q = 1.0), Fusobacteria (W = 16; p = 0.30; q = 1); Actinobacteria (W = 40; p = 0.08; q = 0.45); Epsilonbacteraeota (W = 29; p = 0.67; q = 1.0). B Beta Diversity: RDA plot derived from unweighted UniFrac constrained on host status (Unifrac; PERMANOVA; R2 = 0.06; p = 0.48). The percentage of variance explained by each PC is displayed in descending order after the constrained axis (orange bar). Host stat [file pone.0268730.s002.tif]

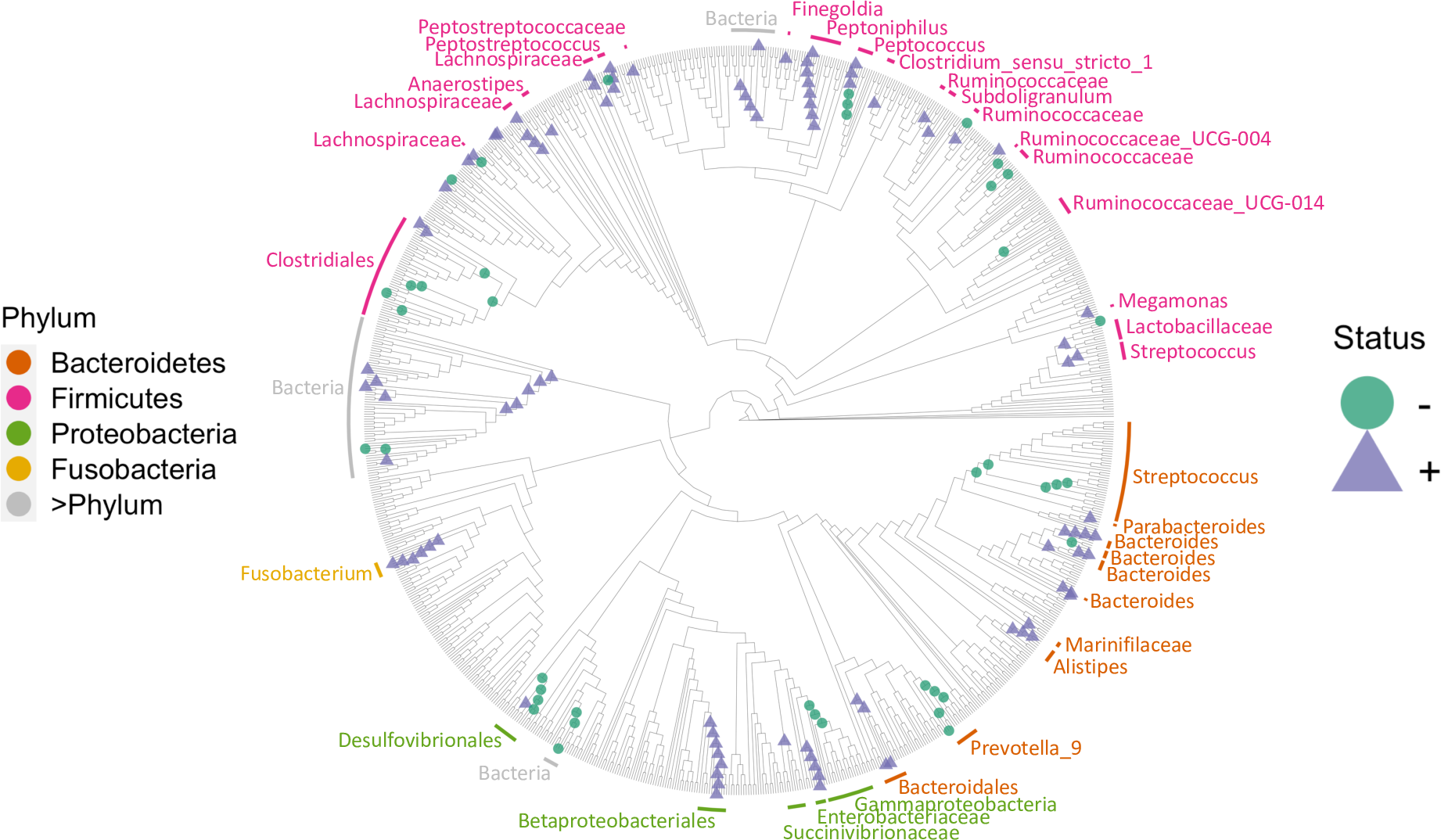

Supplement: S2 Fig — Each microbial feature was modeled as a function of clinical signs. Features of the gut microbiome which significantly associated with the presence (purple triangles) or absence (green circles) of clinical signs are indicated by tree node color (q < 0.05). Phylogenetic tree labels indicate the significant microbial feature’s phylum-level taxonomic label. (TIF) [file pone.0268730.s003.tif]

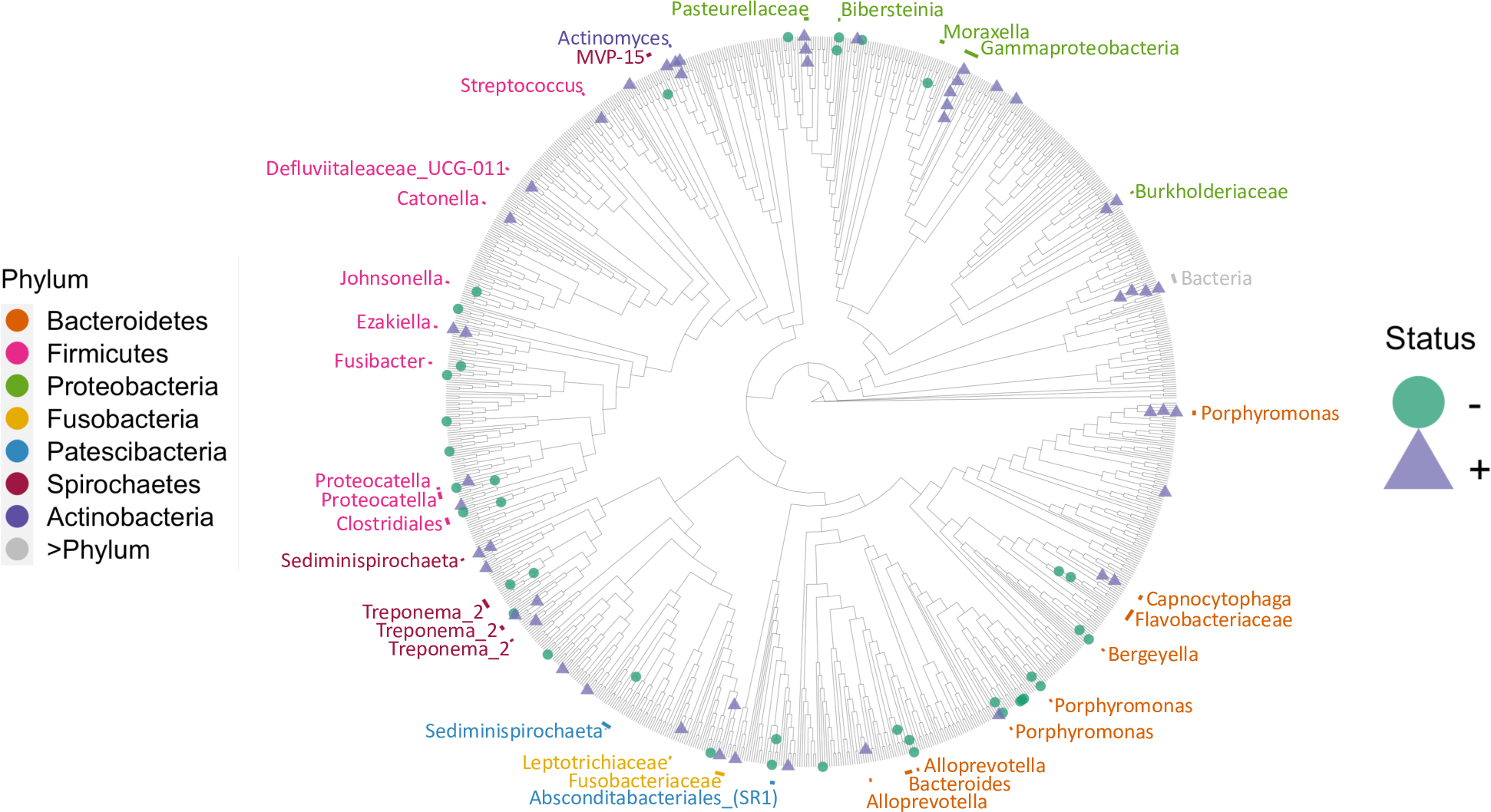

Supplement: S3 Fig — Each microbial feature was modeled as a function of clinical signs. Features of the nasal microbiome which significantly associated with the presence (purple triangles) or absence (green circles) of clinical signs are indicated by tree node color (q < 0.05). Phylogenetic tree labels indicate the significant microbial feature’s phylum-level taxonomic label. (TIF) [file pone.0268730.s004.tif]
